# Rare Paget’s Disease Associated With Breast Cancer in a 70-Year-Old Male

**DOI:** 10.7759/cureus.38225

**Published:** 2023-04-27

**Authors:** Megan Bradley, Brittany Miles, Peter Young, Jing He, Quan D Nguyen

**Affiliations:** 1 Radiology, University of Texas Medical Branch, Galveston, USA; 2 Pathology, University of Texas Medical Branch, Galveston, USA; 3 Radiology, Baylor College of Medicine, Houston, USA

**Keywords:** radiology, management, ultrasound, mammography, male breast cancer, breast cancer, paget's disease of the breast

## Abstract

Male breast cancer is far less common compared to female breast cancer. Paget’s disease of the breast (PDB) is a rare disease, making it even rarer in men. It often presents with eczematous patches over the nipple and areola region, mimics benign dermatological conditions, and can result in a greatly delayed diagnosis. This report presents a rare case of PDB in a 70-year-old male and includes a review of its clinical presentation, radiographic findings, histology, carcinogenic potential, and management.

## Introduction

Male breast cancer accounts for less than 1% when compared to female breast cancer incidence [1]. Paget’s disease of the breast (PDB) is malignant infiltration of the nipple epidermis and accounts for only 1-4.3% of all breast carcinomas [2]. PDB clinically presents with eczematous changes to the nipple-areolar complex, which may appear as reddening, keratosis, or scaling; this may appear extremely similar to benign dermatological conditions. The failure to exclude PDB may lead to misdiagnosis and treatment delays. Patients presenting with dermatological changes to the nipple-areolar complex should receive a complete diagnostic work-up which includes a physical exam, radiographic imaging, and potential histologic confirmation to avoid misdiagnosis. Here, we present a rare case of PDB in a male patient with breast cancer.

## Case presentation

A 70-year-old man, with a strong family history of breast cancer in both his mother and two maternal aunts, presented to his dermatologist with right breast pain and nipple retraction. His dermatologist noted skin induration without palpable masses or bloody discharge. The patient promptly underwent a punch biopsy, which showed invasive ductal carcinoma of the breast with epidermal involvement that was estrogen receptor positive (ER+), progesterone receptor positive (PR+), and human epidermal growth factor receptor 2 positive (HER2+). The patient was then referred for further radiographic workup including mammography, ultrasound, and FDG-PET/CT scan.

**Figure 1:**
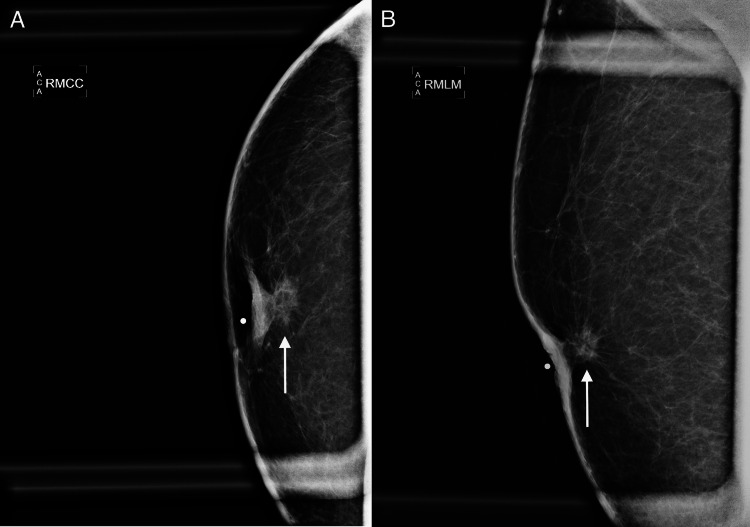
Mammography of the right breast demonstrates a 1.2 cm high density and irregular mass with spiculated margins and associated skin thickening and nipple retraction in the retroareolar region. (A) The magnified craniocaudal (MCC) and (B) magnified mediolateral (MML) views are presented.

**Figure 2:**
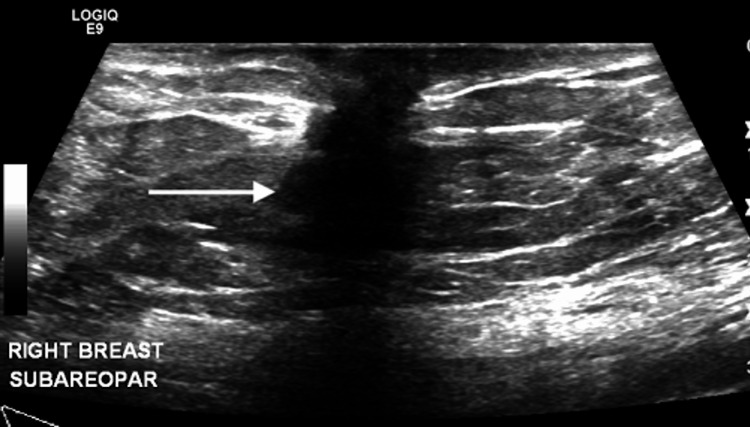
Survey ultrasound of the right breast and axilla demonstrates a 1.4 x 1.3 x 1.2 cm solid, hypoechoic, and irregular mass at the subareolar region. The remainder of the breast and axilla examination is unremarkable.

**Figure 3:**
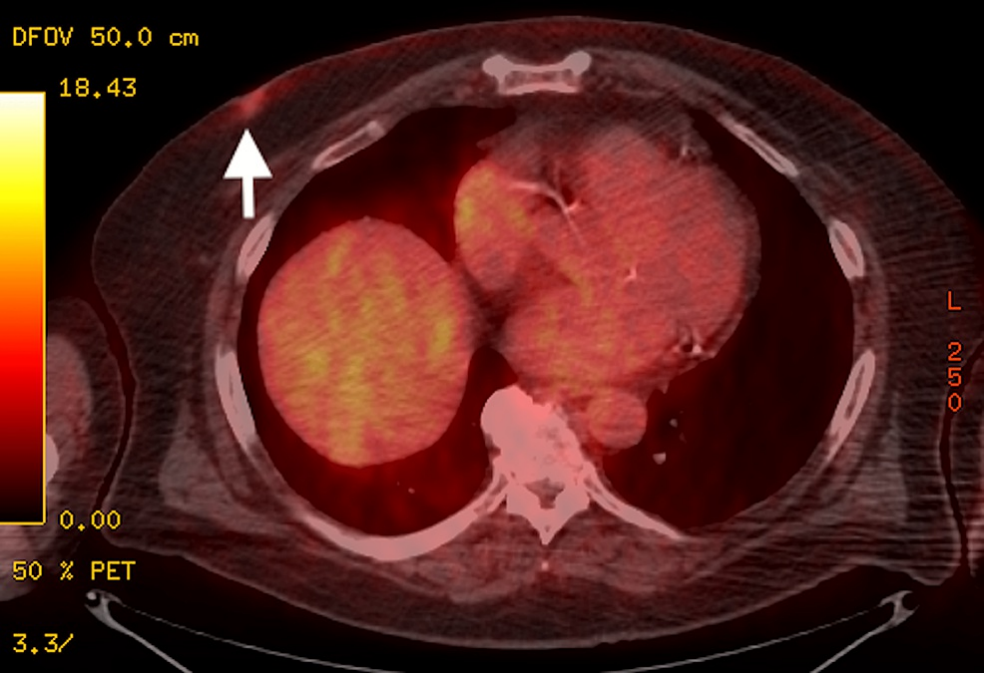
FDG-PET/CT demonstrates mildly hypermetabolic activity with an SUV max of 2.0 at the right subareolar region, which correlates with the known malignancy. No evidence of hypermetabolic nodal or metastatic disease is visualized. FDG: fluorodeoxyglucose; PET: positron emission tomography; SUV: standardized uptake value

Mammography demonstrated a high density and irregularly shaped mass with spiculated margins in the retroareolar region with associated nipple and skin retraction (Figure 1). The ultrasound showed a 1.4 cm solid, hypoechoic, and irregular mass at the retroareolar region (Figure 2). A staging fluorodeoxyglucose (FDG)-positron emission tomography (PET)/CT scan did not show nodal or distant metastatic disease (Figure 3). The lesion was characterized as Breast Imaging Reporting and Data System (BI-RADS) category 6 (biopsy-proven malignancy), and surgical resection was recommended.

**Figure 4:**
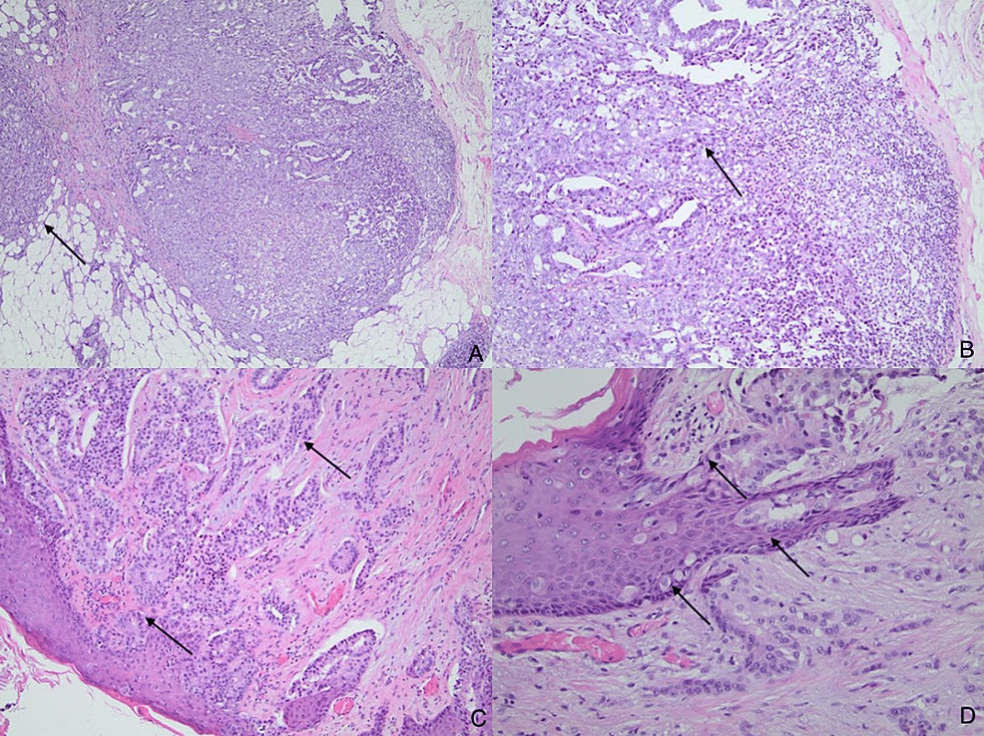
Histological findings of right breast modified radical mastectomy. (A): Hematoxylin and eosin (H&E) staining of the breast mass lesion reveals an invasive ductal carcinoma infiltrating into the adipose tissue (magnification 40x); (B): Excision biopsy of the sentinel lymph node shows metastatic breast ductal carcinoma (magnification 100x); (C): Invasive breast ductal carcinoma involving the dermis and epidermis (magnification 100x); (D): Histologic section of the nipple-areolar skin demonstrates single or clusters of tumor cells spread through the epidermis. The tumor cells demonstrate abundant pale cytoplasm and prominent nucleoli. The histological features are consistent with Paget's disease (magnification 200x)

The patient underwent a modified radical mastectomy. The sentinel lymph node biopsy was positive for metastatic carcinoma and a full axillary dissection was performed. The final pathology report described a 19 mm invasive ductal carcinoma with clear margins. The tumor was grade 2 and was ER+, PR+, and HER2 1+ amplified. Nodal disease with 2mm of an extracapsular extension was present in one of the 15 lymph nodes without perineural or lymphovascular space invasion (Figure 4). The final breast cancer stage was determined to be Stage IIA (pT1cN1aM0).

The patient then underwent adjuvant chemoradiation therapy and subsequent estrogen blockade with tamoxifen. His subsequent mammograms have all been negative for malignancy.

## Discussion

PDB is typically located in the terminal lactiferous ducts and spreads contiguously into the skin of the nipple [3]. It presents with eczematous changes to the nipple-areolar complex and associated symptoms of pruritus, burning, discharge, bleeding, redness, and scaling [4]. The eczematous presentation may result in difficulty differentiating this disease from benign dermatological conditions and may lead to delays in diagnosis of more than six months [5]. Due to the rarity of presentation, especially in male patients, proper diagnosis of PDB is imperative. Over 90% of cases are associated with underlying carcinoma in situ or invasive breast carcinoma [6].

A surgical biopsy is the gold standard for diagnosis. Follow-up diagnostic mammography and ultrasound should be promptly performed to screen for underlying carcinomas [7]. The histological hallmark of PDB is the presence of Paget cells, which are intraepithelial adenocarcinoma cells that often show a clear and vacuolated cytoplasm [4].

Two postulated theories have been established to explain the histogenesis of PDB. The first and more widely supported theory is the epidermotropic theory [3], which states that ductal cells migrate to the nipple-areolar complex through the ductal system [4]. This theory is supported by the presentation or detection of a breast mass [8]. The second theory is the in situ malignant transformation hypothesis, which proposes that PDB develops from keratinocytes independently of an underlying carcinoma [4]. This theory is supported by patients who did not present with a breast mass [8]. 

Molecular subtypes of invasive carcinoma are clinically important because of their association with different risk factors, prognoses, and treatments [9]. These are well established for invasive carcinoma of the breast and include the luminal type A subtype (ER+ or PR+, HER- and Ki-67 index <15%), luminal type B subtype (ER+ or PR+ and HER+, or Ki-67 index >15%), HER2 subtype (ER- and PR-, HER2+), and triple-negative subtype (ER-, PR-, and HER-) [9]. One study from Watcher et al. finds that these molecular subtypes are also identified in both PDB and in PDB with associated invasive carcinoma of the breast (ICB) and ductal carcinoma in situ (DCIS). In PDB, the HER2 subtype was the most common (65.8%), followed by the luminal B subtype (28.9%), then the triple-negative subtype (5.3%), with no luminal type A subtype present. In PDB with associated ICB, the luminal type B subtype was the most common (50%), followed by the HER2 subtype (40%), then the triple-negative subtype (10%), with no luminal type A subtype present. This is in contrast to ICB, in which the luminal type B subtype is by far the most common (70%), followed by the HER2 subtype (15%) and the triple-negative subtype (15%), with no luminal type A subtype present.

Male breast cancer often presents at a later stage when compared to females; due to this later presentation, approximately 40% of male breast cancer patients present at stage III or IV [10], requiring prompt treatment. No randomized control studies comparing treatment options for males with PDB with associated ICB have been performed, which greatly limits evidence-based clinical practice for the best course of treatment [11]. However, mastectomy with axillary lymph node biopsy for staging is considered the standard treatment for PDB [11]. In addition, while there is no current data that hormone therapies such as tamoxifen reduce the recurrence risk in patients with PDB without an underlying carcinoma [4], this patient had an underlying ICB that was ER+, so tamoxifen was added to the treatment plan.

## Conclusions

Male breast cancer and PDB disease are both extremely rare. PDB must be considered in this patient population who presents with eczematous changes at the nipple-areolar complex to not delay diagnosis. Biopsy should be performed promptly and followed by further radiographic studies including mammography and ultrasonography to exclude underlying malignancy. Surgical excision is the treatment of choice, and hormonal therapy is often added due to positive hormonal receptors. We hope this case brings further attention to the rarity of male breast cancer and PDB.
